# The Effect of Spiritual Well-Being on Alternative Treatment Attitudes: A Descriptive Study of Patients with Type 2 Diabetes in Turkiye

**DOI:** 10.1007/s10943-025-02320-8

**Published:** 2025-05-30

**Authors:** Canan Güngör, Rukiye Burucu

**Affiliations:** 1 Selçuklu District Directorate of Health, Mümtaz Koru Tuberculosis Dispensary, Konya, Türkiye; 2 Seydişehir Kamil Akkanat Faculty of Health Sciences, Faculty Member of Internal Medicine Nursing, Necmettin Erbakan University, Seydişehir, Konya, Türkiye

**Keywords:** Type 2 diabetes, Complementary and alternative therapies, Spiritual well-being, Nursing

## Abstract

This paper investigated the effect of spiritual well-being on attitudes toward complementary and alternative medicine. This descriptive and correlational study was conducted in a university hospital in Turkiye. The study population consisted of all patients with type 2 diabetes treated in the hospital. The sample consisted of 250 participants. The sample size was calculated based on the regression results reported by Ben-Arye et al. (2011). Data were collected using a personal information form, the Holistic Complementary and Alternative Medicine Questionnaire and the Three-Factor Spiritual Well-Being Scale. Participants had a mean age of 57.10 ± 6.758 years. They had a mean Body Mass Index of 30.08 ± 4.110. Most patients with type 2 diabetes use alternative methods, such as massage and thyme. There is no correlation between spiritual well-being and Holistic Complementary and Alternative Medicine attitudes. Healthcare professionals should integrate modern and alternative diabetes treatment methods. Patients trained by diabetes education nurses are less likely to make wrong choices about Holistic Complementary and Alternative Medicine.

## Introduction

Diabetes is a major public health issue (WHO, 2013). More than 95% of people with diabetes have type 2 diabetes (Mumcu & İnkaya, 2020). The number of individuals with diabetes is increasing day by day (WHO, 2024). In addition to medical treatment, other alternative methods are used for the treatment of diabetes (Abu-Odeh & Talib, 2021). One of the factors that are effective in managing the disease is the individual’s spirituality (Celik et al., 2022).

### Diabetes and Alternative Treatment

People in poor health turn to complementary and alternative medicine (CAM) (Eleazu et al., 2021). Alternative therapies are proven or unproven methods in place of standard medical treatments. Complementary therapies are used in combination with standard treatments (AmericanCancerSociety, 2021). People with diabetes also turn to CAM, such as acupuncture, mind–body therapies, spiritual therapies, balneotherapy, homeopathy, biofeedback, meditation, chelation, massage, energy therapy, breathing exercises, leeches, yoga, music therapy, prayer, relaxation techniques, cupping, honey (Alzahrani et al., 2021), herbal medicines, diet, etc. (Ismail et al., 2019). Turkish people with type 2 diabetes use black salsify, cinnamon, goldenseal, laurel, yarrow, Jerusalem sage, giyamambel, thyme, and maldili (baresaspi) (Abu-Odeh & Talib, 2021). Researchers maintain that more research is warranted on this topic (Alzahrani et al., 2021).

### Diabetes and Spirituality

Spirituality, as well as medical treatments, helps patients feel better (Erişen & Sivrikaya, 2017). Spirituality means “spiritual things,” “the strength of the heart,” or “morale” (Turkish Language Association, 2022). Spirituality is related to goals, values, and beliefs. It is a universal experience in which one feels connected to a higher power (Ekşi & Kardaş, 2017; Weathers, 2019). Healthcare professionals should be aware of the spiritual well-being of their patients because spirituality contributes significantly to health (Weathers, 2019). Celik et al. (2022) investigated the effect of religious coping on disease acceptance and management among Turkish patients with diabetes and found that patients who felt spiritually more had better glycemic parameters (Celik et al., 2022).

In general, diabetic patients with high spirituality better cope with the disease, perform self-care and adapt to novel situations (Faghani et al., 2018). There is a large body of research on the popularity of CAM among patients with diabetes (Abu-Odeh & Talib, 2021; Alzahrani et al., 2021; Can Çiçek et al., 2021; Chandra et al., 2020; Eleazu et al., 2021; Gall et al., 2021; Ismail et al., 2019; Kılıç et al., 2020; Satıl & Açar, 2020). Some researchers have also addressed the spiritual aspects of patients with diabetes (Choi & Hastings, 2019; Faghani et al., 2018; Javanmardifard et al., 2020). However, no researchers have ever focused on the effect of spiritual well-being on the CAM preferences of Turkish patients with diabetes. We think this paper will contribute to the literature because it is the first study to investigate the effect of spiritual well-being on the CAM preferences of Turkish people with type 2 diabetes.

## Method

This was a descriptive and correlation study.

### Population and Sample

This study was conducted in a university hospital. The study population consisted of all patients with type 2 diabetes treated in the hospital. The sample size was calculated based on the regression results reported by Ben-Arye et al. (2011), who explored the association between spiritual perspectives and complementary medicine use among patients with Type 2 diabetes in Israel. The researchers conducted logistic regression concerning CAM use among religious (B:1.300 and p:0.020) and traditional people (B:0.341 and p:0.300). The regression analysis involved three parameters (Ben-Arye et al., 2011). In accordance with the literature (Bujang et al., 2018) we set one hundred as the base number and added 50 more people for each parameter in the logistic regression (100 + 150). Thus, the sample size was calculated to be at least 250. Data were collected using a personal information form, the Holistic Complementary and Alternative Medicine Questionnaire (HCAMQ), and the Three-Factor Spiritual Well-Being Scale (SWBS).

The following are the research questions:

1. What HCAMQ scores do participants have?
2. Do participants use CAM?
3. What type of CAM do participants use?
4. Is there a correlation between SWBS and HCAMQ scores?

The inclusion criteria were (1) having had diabetes for at least a year, (2) being 18 to 65 years of age, and (3) volunteering. The exclusion criteria were (1) being pregnant and (2) having communication and cognitive issues.

### Data Collection

The data were collected face-to-face in the diabetes training outpatient clinic of the hospital. The data were collected using a personal information form, the Holistic Complementary and Alternative Medicine Questionnaire (HCAMQ), and the Three-Factor Spiritual Well-Being Scale (SWBS).

### Personal Information Form

The personal information form was developed by the researchers (Can Çiçek et al., 2021; Choi & Hastings, 2019; Eleazu et al., 2021; Faghani et al., 2018; Gall et al., 2021; Ismail et al., 2019; Javanmardifard et al., 2020). The form consisted of 25 items on sociodemographic characteristics, CAM preferences, and diabetes and treatment.

### Holistic Complementary and Alternative Medicine Questionnaire

The Holistic Complementary and Alternative Medicine Questionnaire (HCAMQ) was developed by Hyland et al. (2003) (Hyland et al., 2003) and adapted to Turkish by Erci (2007). The questionnaire consists of 11 items rated on a six-point Likert-type scale [(1) strongly agree, (2) agree, (3) somewhat agree, (4) somewhat disagree, (5) disagree, (6) strongly disagree)]. The questionnaire has two subscales: CAM (Items 2, 4, 6, 8, 9, and 11) and holistic health (HH) (1, 3, 5, 7, and 10). The “CAM” subscale relates to the respondent’s beliefs about the scientific validity of CAM. The “holistic health” subscale relates to the respondent’s perception of individuality in his/her care. Four items are reverse-scored (Items 2, 4, 6, and 9). The total score ranges from 11 to 66, with lower scores indicating more positive attitudes toward CAM. The HCAMQ-TR has a Cronbach’s alpha of 0.72 (Erci, 2007), which was 0.68 in the present study. The “CAM” and “HH” subscales have Cronbach’s alpha scores of 0.817 and 0.41, respectively.

### Three-Factor Spiritual Well-Being Scale

The Three-Factor Spiritual Well-Being Scale (TF-SWBS) was developed by Ekşi and Kardaş (2017) to identify the processes by which adults understand and live their lives in terms of personal, social, environmental, and transcendental aspects in line with their values and ultimate meanings. The Spiritual Well-Being Scale was an earlier version (Ekşi & Kardaş, 2017). The researchers conducted a confirmatory factor analysis, which revealed a three-factor structure (Kardaş, 2019). The instrument consists of 29 items rated on a five-point Likert-type scale [(1 = Not applicable to me at all, 2 = Not applicable to me, 3 = Somewhat applicable to me, 4 = Quite applicable to me, 5 = Completely applicable to me)]. The instrument has three subscales: transcendence (Items 1, 4, 5, 8, 9, 12, 13, 16, 17, 20, 21, 24, 25, 27, and 29), harmony with nature (Items 2, 6, 10, 14, 18, 22, and 28), and anomie (Items 3, 7, 11, 15, 19, 23, and 26). The items of the subscale “anomie” are reverse-scored. The total score ranges from 29 to 145, with higher scores indicating higher levels of spiritual well-being. The instrument has a Cronbach’s alpha of 0.886 (Ekşi & Kardaş, 2017), which was 0.923 in the present study. The subscales “transcendence,” “harmony with nature,” and “anomie” have Cronbach’s alpha scores of 0.845, 0.733, and 0.752, respectively.

Koenig and El Zaben (2021) suggest that in the process of developing a scale with spirituality content; pretesting the scale items, then applying the scale to the target group, reducing the items by removing the items deemed necessary, and then determining the subscales. (Koenig & Al Zaben, 2021). It is explained that TF_SWBS follows these rules (Ekşi & Kardaş, 2017). Researchers emphasize that scales contaminated with mental health indicators should not be used in religion and spirituality research. Koenig and Carey make suggestions regarding the use of contaminated scales. One of them is that if the scale is contaminated, the dependent variable should be the score of the scale measuring health. In this case, the independent variable should be the score indicating spiritual well-being (Koenig & Carey, 2024). Another suggestion is to report the subscale scores separately, not to report the total score and to look at the mediating role (Koenig & Carey, 2025).

Since this study was not a validity and reliability study, no item was removed. SWBS subscale scores were determined as independent variables. In addition, the total score was not evaluated, but subscale scores were analyzed separately. Since the prerequisites for examining the mediation role were not met, it was decided that there was no mediating effect and therefore the mediating role could not be examined.

The dependent variable was the participants’ attitudes toward CAM. The independent variables were spiritual well-being, age, gender, education, employment status, income, tobacco use, receiving training in DM, having diabetes-related health problems, the need for information due to diabetes, and being on medication for diabetes.

### Ethical Considerations

The study was approved by the health sciences ethics committee of XXX University (Date: 16.10.2021 & No: 2021/14–77). Permission was obtained from the hospital (E-14567952–900-108215). The research adhered to the Strengthening the Reporting of Observational Studies in Epidemiology (STROBE) guidelines (Equador, 2019). The study was conducted according to the ethical principles outlined by the World Medical Association’s Declaration of Helsinki (Türk Tabipler Birliği, 2013). All patients were briefed about the research purpose and procedure. Informed consent was obtained from those who agreed to participate.

### Analysis

The data were analyzed using the Statistical Package for Social Science (SPSS, IBM v. 25). Skewness and kurtosis values (-1 + 1) were analyzed for normality testing (George & Mallery, 2019). Gender, diagnosis time, comorbidities, having DM-related problems, receiving training in DM, the source of training, the source of information, employment status, perceived health, the type of medication for DM, the type of herbal products used, BMI, SWBS subscale, and HCAMQ total and subscale scores were normally distributed. The other variables were nonnormally distributed. The nonnormally distributed data were given as medians (1st-3rd quartile) in the table. Numbers, means, and standard deviations were used for sociodemographic and disease-related characteristics. The independent-sample t-test and one-way analysis of variance (ANOVA) were used for normally distributed data, while the Mann–Whitney U and Kruskall-Wallis tests were used for nonnormally distributed data. Fisher’s Least Significant Difference (LSD) test was used for post hoc pairwise comparisons to determine the source of significant differences. Pearson’s and Spearman’s correlations were used to determine the relationship between variables. A linear regression analysis was performed for the appropriate variables. It was done by an expert with analysis.

## Results

Participants had a mean age of 57.10 ± 6.758 years. They had a mean Body Mass Index (BMI) of 30.08 ± 4.110. They had a mean SWBS total score of 30.72 ± 7.197. They had mean SWBS “transcendence,” “anomie,” and “harmony with nature” subscale scores of 40.77 ± 11.766, 19.08 ± 6.271, and 19.68 ± 5.923, respectively. They had a mean HCAMQ score of 30.72 ± 7.197 (Table 1).

**Table 1:** Numerical data

Variables	Min- Max	Mean	SD
Age (year)	32–65	57.10	6.758
BMI	20–45	30.08	4.110
SWBS transcendence	20–75	40.77	11.766
SWBS anomie	8–35	19.08	6.271
SWBS harmony with nature	7–35	19.68	5.923
HCAMQ total	17–53	30.72	7.197
HCAMQ holistic health	7–24	12.94	3.024
HCAMQ complementary and alternative medicine	7–33	17.78	5.848

*BMI:* Body mass index, *SWBS:* Spiritual Well-Being, *HCAMQ:* Holistic Complementary and Alternative Medicine Questionnaire

More than half of the participants were men (54%). Most participants were married (82.4%). The majority of the participants had primary school degrees (77.2%). Most participants had a negative income (income < expense) (89.2%). More than half of the participants had comorbidities (68.8%). Over than half of the participants had DM-related health problems (61.6%). Most participants did not want to use medications for DM (91.6%). More than half of the participants had received training in DM before (52.8%). Less than half of the participants had received training in DM from nurses (42.4%). Most participants preferred to use CAM (81.2%). A quarter of the participants used thyme (26.4%). More than a quarter of the participants were diagnosed with DM 6–10 years ago (32.4%). More than half of the participants were on insulin medication (60.4%). Less than half of the participants believed in the effectiveness of the treatment (45.2%). Participants who needed other patients to learn about their disease had significantly lower mean HCAMQ total and subscale scores than others. Participants who had never received training in DM had a significantly higher mean HCAMQ “HH” subscale score than others. Participants with a low income had a significantly higher mean HCAMQ “HH” subscale score than others. Participants who were on oral antidiabetic drugs (OADs) had significantly lower mean HCAMQ total and “CAM” subscale scores than those who were not (p < 0.05) (Table 2).

**Table 2:** The distribution of HCAMQ scores by independent variables *(n:250)*

		*n*	%	HCAMQ HH	HCAMQ CAM	HCAMQ Total
				Mean ± SD	Mean ± SD	Mean ± SD
Gender	Man	115	46.0	12.88 ± 3.304	18.37 ± 5.942	31.24 ± 7.580
	Woman	135	54.0	12.99 ± 2.775 t = 1.125 p = 0.781	17.29 ± 5.742 t = 0.014 p = 0.147	30.27 ± 6.852 t = 0.065 p = 0.289
Do you have comorbidities?	Yes	172	68.8	12.93 ± 3.176	17.84 ± 6.043	30.77 ± 7.406
	No	78	31.2	12.95 ± 2.677 t = 4.402 p = 0.964	17.67 ± 5.476 t = 3.002 p = 0.831	30.62 ± 6.760 t = 0.527 p = 0.877
Have you ever received training in DM?	Yes	132	52.8	13.61 ± 3.113	18.23 ± 5.647	31.84 ± 6.925
	No	118	47.2	12.19 ± 2.745 t = 3.671 **p = 0.000**	17.28 ± 6.048 t = 1.764 p = 0.200	29.47 ± 7.318 t = 1.796 **p = 0.009**
Do you have DM-related health problems?	Yes	154	61.6	12.86 ± 2.829	17.78 ± 5.855	30.64 ± 6.921
	No	96	38.4	13.05 ± 3.326 t = 4.339 p = 0.633	17.79 ± 5.867 t = 0.009 p = 0.987	30.84 ± 7.655 t = 925 p = 0.831
Employment status	Paid employee	38	15.2	13.45 ± 2.984	17.47 ± 5.087	30.92 ± 6.524
	Retired	89	35.6	13.12 ± 3.257	18.35 ± 5.912	31.47 ± 7.568
	Unemployed	123	49.2	12.64 ± 2.849 F = 1.298 p = 0.275	17.47 ± 6.030 F = 0.642 p = 0.527	30.11 ± 7.121 F = 0.936 p = 0.0.393
How is your health?	Bad	55	22.0	12.58 ± 2.872	16.85 ± 5.914	29.44 ± 7.231
	Okay	133	53.2	12.99 ± 2.891	17.95 ± 5.519	30.94 ± 6.497
	Good	62	24.8	13.13 ± 3.433 F = 0.525 p = 0.592	18.26 ± 6.453 F = 950 p = 0.388	31.39 ± 8.472 F = 1.205 p = 0.301
What type of medication are you on for DM?	OADs^a^	17	6.8	12.59 ± 1243	13.65 ± 5.968	26.24 ± 8.460
	Insulin^b^	151	60.4	12.87 ± 3.135	18.03 ± 5.701	30.90 ± 7.194
	OADs + Insulin^c^	82	32.8	13.12 ± 2.813 F = 0.297 p = 0.743	18.20 ± 5.832 F = 4.724 **p = 0.010**	31.32 ± 6.679 F = 3.709 **p = 0.026**
Do you believe in the effectiveness of your treatment?	Yes	109	43.6	13.28 ± 3.389	18.50 ± 5.840	31.78 ± 7.679
	Somewhat yes	113	45.2	12.70 ± 2.853	17.05 ± 5.704	29.75 ± 6.726
	No	28	11.2	12.54 ± 1.934 F = 1.319 p = 0.269	17.96 ± 6.298 F = 1.712 p = 0.183	30.50 ± 6.758 F = 2.238 p = 0.109
When were you diagnosed with DM?	1–2 years ago	19	7.6	13.26 ± 3.842	20.05 ± 2.738	33.32 ± 4.137
	3–5 years ago	35	14.0	12.46 ± 2.593	19.00 ± 6.226	31.46 ± 6.879
	6–10 years ago	81	32.4	13.52 ± 2.838	18.02 ± 6.150	31.54 ± 7.468
	11–15 years ago	59	23.6	12.63 ± 3.022	17.22 ± 5.654	29.85 ± 7.095
	16–20 years ago	56	22.4	12.61 ± 3.189 F = 1.354 p = 0.251	16.50 ± 5.902 F = 1.970 p = 0.100	29.11 ± 7.636 F = 1.922 p = 0.107
What herbal products do you consume?	Black salsify	12	4.8	13.58 ± 3.204	16.50 ± 5.054	30.08 ± 5.992
	Cinnamon	42	16.8	14.14 ± 4.100	19.62 ± 6.188	33.76 ± 9.239
	Ceterach	12	4.8	12.67 ± 3.085	18.83 ± 3.380	31.50 ± 4.834
	Laurel	35	14.0	12.37 ± 2.315	16.54 ± 5.321	28.91 ± 5.643
	Pennyroyal	13	5.2	12.00 ± 2.082	16.08 ± 6.144	28.08 ± 6.576
	Jerusalem sage	6	2.4	13.33 ± 2.422	15.50 ± 4.848	28.83 ± 6.616
	Gıyamambel	8	3.2	13.00 ± 2.138	20.75 ± 3.240	33.75 ± 3.808
	Thyme	66	26.4	13.00 ± 2.660	17.15 ± 6.153	30.15 ± 7.269
	Maldili	9	3.6	12.89 ± 2.369	21.00 ± 3.041	33.89 ± 3.919
	None	47	18.8	12.30 ± 3.162 F = 1.354 p = 0.210	17.66 ± 6.441 F = 1.622 p = 0.110	29.96 ± 7.265 F = 1.857 p = 0.057
Who gave you training in DM?	Doctors^c^	19	7.6	12.53 ± 3.306	18.53 ± 6.077	31.05 ± 6.240
	Nurses^d^	106	42.4	13.93 ± 3.362	18.64 ± 5.586	32.58 ± 7.329
	Other healthcare professionals^b^	7	2.8	13.57 ± 1.718	14.14 ± 4.298	27.71 ± 5.559
	No one^a^	118	47.2	12.07 ± 2.403 F = 7.919 **p = 0.000**	17.11 ± 6.011 F = 2.325 p = 0.075	19.18 ± 6.956 F = 4.783 **p = 0.003**
To whom do you ask for information?	Doctors^e^	13	5.2	11.54 ± 3.597	22.15 ± 5.178	33.69 ± 5.736
	Diabetes nurses^d^	81	32.4	13.89 ± 3.082	17.52 ± 5.868	31.41 ± 7.564
	Other healthcare professionals^f^	20	8.0	14.65 ± 3.031	21.10 ± 5.190	35.75 ± 6.668
	Relatives and friends^b^	34	13.6	12.35 ± 1.889	16.97 ± 5.254	29.32 ± 5.244
	Other patients^a^	55	22.0	11.47 ± 2.742	16.47 ± 6.301	28.15 ± 7.619
	Internet^c^	47	18.8	12.85 ± 2.971 F = 6.219 **p = 0.000**	17.74 ± 5.277 F = 3.640 **p = 0.003**	30.60 ± 6.500 F = 4.508 **p = 0.001**
What DM-related health problems do you have?	Hypoglycemia	44	17.6	12.61 ± 2.935	17.20 ± 6.148	29.82 ± 7.683
	Hyperglycemia	77	30.8	13.53 ± 2.431	18.31 ± 5.990	31.84 ± 6.972
	Foot scars	30	12.0	11.20 ± 2.140	17.10 ± 4.880	28.30 ± 4.935
	Cardiovascular problems	21	8.4	13.48 ± 3.26	17.90 ± 5.449	31.38 ± 6.376
	None	78	31.2	13.05 ± 3.556 F = 0.229 p = 0.633	17.82 ± 6.060 F = 0.000 p = 0.987	30.72 ± 7.197 F = 0.046 p = 0.831
				**Median (1st-3rd quartile**	**Median (1st-3rd quartile**	**Median (1st-3rd quartile**
Do you smoke?	Yes	44	17.6	12.00 (10.00–16.009	19.50 (13.25–22.75)	31.50 (27.25- 34.00)
	No	206	82.4	13.00 (11.00–15.00) U:1853.500 p:0.281	17.00 (12.00–23.00) U:1853.500 p:0.281	31.00 (25.00–35.00) U:1853.500 p:0.281
Marital status	Married	206	82.4	12.00 (11.00–15.00)	18.00 (13.00–23.00)	32.00 (25.00–35.00)
	Single	44	17.6	13.00 (11.25–15.00) U:4150.000 p:0.378	15.00 (12.00–20.00) U:3769.000 p:0.079	29.00 (24.25–33.00) U:3928.000 p:0.165
Do you use alternative products?	Yes	203	81.2	13.00 (11.00–15.00)	18.00 (13.00–23.00)	31.00 (25.00–35.00)
	No	47	18.8	12.00 (10.00–15.00) U:4244.500 p:0.236	16.00 (12.00–23.00) U:4634.500 p:0.760	32.0023.00–33.00) U:4774.00 p:0.374
Do you need information about DM?	Yes	55	78.0	14.00 (11.00–16.00)	18.00 (14.00–23.00)	32.00 (27.00–36.00)
	No	195	22.0	12.00 (10.00–14.00) U:5061.500 p:0.523	17.00 (12.00–22.00) U:4790.00 p:0.226	30.00 (23.00–34.00) U:5033.00 p:0.486
Do you want to use your medication?	Yes	19	7.6	14.00 (12.00–14.00)	17.00 (12.00–24.00)	31.00 (23.00–38.00)
	No	229	91.6	13.00 (11.00–15.00) U:1853.500 p:0.281	18.00 (13.00–23.00) U:2066.000 p:0.715	31.00 (25.00–35.00) U:2032.000 p:0.633
Education (degree)	Primary school	193	77.2	12.00 (11.00–15.00)	18.00 (12.00–23.00)	31.00 (25.00–35.00)
	Middle school	40	16.0	13.00 (10.00–14.00)	16.50 (12.25–22.75)	30.50 (25.00–34.75)
	Bachelor’s or higher	17	6.8	15.00 (10.50–17.00) KW:3.920 p:0.141	18.00 (15.50–22.00) KW:0.574 p:0.750	33.00 (30.50–34.50) KW:2.063 p:0.357
Living arrangement	Family	225	90.0	13.00 (11.00–15.00)	17.00 (12.50–22.00)	31.00 (25.00–35.00)
	Friends	6	2.4	11.00 (9.75–13.00)	23.0017.00–24.25)	32.50 (29.75–35.00)
	Alone	19	7.6	13.00 (12.00–15.00) KW:2.864 p:0.239	21.00 (12.00–24.00) KW:3.601 p:0.165	31.00 (27.00–39.00) KW:1.717 p:0.424
Income	Income < expense^a^	223	89.2	13.00 (11.00–15.00)	18.00 (12.00–22.00)	31.00 (25.00–34.00)
	Income = expense^b^	18	7.2	12.00 (11.00–15.50)	21.50 (12.00–25.25)	32.00 (23.00–40.00)
	Income > expense^c^	9	3.6	15.00 (14.00–17.00) KW:7.582 **p:0.023**	19.00 (14.00–25.50) KW:2.047 p:0.359	38.00 (29.00–42.00) KW:3.767 p:0.152
What type of CAM do you use?	Massage	23	9.2	13.00 (11.00–16.00)	18.00 (13.00–21.00)	33.00 (25.00–36.00)
	Sports/Exercise	20	8.0	12.50 (9.50–17.00)	16.00 (12.00–21.75)	30.00 (24.00–35.25)
	Hirudotherapy	16	6.4	14.00 (11.00–16.30)	19.50 (15.25–25.00)	34.00 (28.00–39.75)
	Music therapy	10	4.0	13.00 (12.50–14.00)	20.00 (10.00–21.25)	33.50 (23.00–34.00)
	Other	166	66.4	14.00 (11.00–17.00) KW:5.283 p:0.382	21.00 (15.00–24.00) KW:4.004 p:0.549	32.00 (27.00–39.00) KW:4.907 p:0.427

Bold values indicate statistical significance (*p* < 0.05)

t: Independent Samples t-test. F = One Way Analysis. KW = Kruskall-Wallis U = Mann–Whitney U; LSD; a < b < c < d < e < f, *HCAMQ:* Holistic Complementary and Alternative Medicine Questionnaire, *HH:* Holistic health; *CAM*: Complementary and alternative medicine

There was no correlation between age and BMI and scale scores (p > 0.05) (Table 3).

**Table 3:** Correlations

Variables	HCAMQ Total		HCAMQ HH		HCAMQ CAM	
	r	p	r	p	r	p
Age^a^	0.047	0.460	0.119	0.060	0.008	0.902
BMI^b^	-0.018	0.780	-0.118	0.061	0.39	0.535
SWBS Transcendence^b^	0.092	0.149	0.116	0.066	0.053	0.408
SWBS Harmony with nature^b^	0.089	0.161	0.043	0.496	0.087	0.170
SWBS Anomie^b^	0.025	0.697	0.035	0.582	0.012	0.845

^*^Correlation is significant at the 0.05 level (2-tailed).^**^Correlation is significant at the 0.01 level (2-tailed), ^a^: Spearman’s Correlation, ^b^: Pearson’s Correlation, *HCAMQ:* Holistic Complementary and Alternative Medicine Questionnaire, *HH:* Holistic health; *CAM*: Complementary and alternative medicine, *SWBS:* Spiritual Well-Being

As seen in Table 3, in the multiple regression analysis for the HCAMQ total score, “SWBS Transcendence” and “SWBS Harmony with nature” values were not included in the analysis because a high level of correlation was found between them (r > 0.80). The regression model (Enter) created for HCAMQ total score regarding the status of receiving education about DM, the type of treatment used, from whom DM education was received, and how they met their need for information about DM was found to be statistically significant (F = 4.824; p < 0.001). The regression model explained 0.090 of the HCAMQ total score. For the HCAMQ total score, the variables of who meets the need for information about DM (p:0.003) and the type of treatment used (p:0.036) were found significant (p < 0.05). The model explained nine percent of the total variance (Table 4).

**Table 4:** Regression analysis results

	β^1^ (%95 CI)	SE	β^2^	t	p	Zero	Partial	VIF
(Constant)	33,655 (26,628 – 38,682)	2,552		13,186	0,000			
SP Anomi	0,046 (-0,097 – 0,189)	0,073	0,040	0,631	0,529	,025	,040	1,075
Have you ever received training in DM?	− 1,070 (4,008 – 1,868)	1,492	− 0,074	− 0,717	0,474	− ,165	− ,046	2,882
Who gave you training in DM?	− 1,005(–2,326 – 0,317)	0,671	− 0,153	− 1,498	0,135	− ,200	− ,095	2,781
To whom do you ask for information?	− 0,814 (-1,021 – 0,003)	0,269	− 0,187	− 3,021	**0,003**	− ,162	− ,190	1,030
What type of medication are you on for DM?	1,635(0,1107–3,163)	0,776	0,130	2,108	**0,036**	,122	,134	1,027
F = 4,824; p < 0,001; Adj. R^2^ = 0,090; SE of Estimate = 6,936; DW: 1,816								

## Discussion

This study focused on patients with type 2 diabetes and investigated the effect of spiritual well-being on their attitudes toward CAM. Although consuming thyme and receiving massages were our participants’ most common CAM practices, there was no statistically significant difference (p > 0.05). Four out of five participants used CAM (82.1%). Our participants had a moderate level of CAM attitude. Studies show that diabetic individuals have a rate of CAM use > 50% (Almohaileb, 2023; Özkan et al., 2023; Patil et al., 2024). Alternative methods used include herbs, acupuncture, acupressure, massage, mind and body therapies (Parliani & Pearkao, 2024). Nangandu Hikaambo et al. (2022) determined that four in five patients with diabetes used CAM, such as garlic (58.4%), moringa (42.6%) (Nangandu Hikaambo* et al., 2022). Most of our participants consumed thyme, which is popular in Turkiye. Turkish people consume thyme juice for different reasons, one of which is to stabilize blood sugar levels (Başer, 2022). They consume thyme probably because it is easily accessible, familiar, and cheap.

Our participants stated that they received massages. Regular foot massages help heal foot wounds and reduce complaints related to peripheral neuropathy in patients with DM (Sunarmi et al., 2022). Bayat et al. (2020) argue that massages have a positive effect on neuropathy and HbA1 C levels (Bayat et al., 2020). According to the results of the research, in adults with diabetes; Acupuncture point massage has a positive effect on individuals’ quality of life and blood sugar levels, (Lyu et al., 2019), back massage improves sleep quality (Syaripudin et al., 2021), massage of the feet appears to relieve signs of peripheral neuropathy (Rivaz et al., 2021). We did not ask our participants about any specific message. Therefore, they thought of it as a general term and stated they felt relaxed when they had it.

Participants who had never received training in DM had more positive attitudes toward CAM. Participants with a negative income had more positive attitudes toward CAM. Participants who learned about diabetes from other patients had more positive attitudes toward CAM. Participants who were on OADs had more positive attitudes toward CAM. Radwan et al. (2020) argue that diabetic patients with low education levels are more likely to use CAM (Radwan et al., 2020). Kaynak (2017) also found that diabetic patients who had never received training in DM were more likely to use CAM (Kaynak & Polat, 2017). Patients who receive training in DM are better at managing their disease (Rutledge, 2021). Ünal Aslan (2022) determined that two in five diabetic patients who used CAM were on insulin (Unal Aslan, 2022). Based on these findings, we can draw some conclusions. First, diabetes training is critical for diabetic patients. Second, patients who do not receive training in DM turn to different solutions. Third, insulin users are highly likely to receive diabetes training, whereas OAD users do not. Patients who do not receive training in DM turn to different resources to solve their diabetes-related problems.

Diabetic patients who know little about their condition are more likely to use CAM. Family members (42.7%), friends (25%), or social media (17.7%) encourage those patients to turn to CAM. Radwan (2020) reported that only one in ten patients with diabetes was encouraged by healthcare professionals to use CAM (Radwan et al., 2020). Kifle (2021) found that two in five patients with diabetes turned to traditional healers to learn more about their condition (38.8%) (Kifle, 2021). Patients must have access to health care and find fast and accurate solutions to their problems. This is one of the reasons why they use CAM (Kaynak & Polat, 2017). The more affordable and accessible a method is, the more patients use it. Alay et al. (2018) reported that most CAM users had an income of 1000- 2000 TL (Alay et al., 2018). In other words, diabetic patients with a low income are more likely to turn to CAM therapies because they are cheaper than modern medical interventions. This suggests that nurses should be there for diabetic patients and inform them about their disease and its treatment so that they can avoid misusing CAM therapies.

## Limitations

This study has two limitations. First, the SWB scale has three subscales, but two of these three subscales are contaminated with mental health indicators. Therefore he had to consider only the subscales. Second, the research was conducted at only one institution. Therefore, the results are sample specific.

## Conclusion

Patients with diabetes have moderate levels of spiritual well-being and CAM attitudes. Most of the patients with diabetes use CAM. For example, they have massage and consume thyme. There is no relationship between spiritual well-being and CAM attitudes. The way of meeting the individual’s need for information about DM and the type of treatment used affect CAM attitudes. The most important result of this study is that patients who did not receive education about their disease, used oral antidiabetics, did not meet their information needs and tried to meet their information needs from other patients were more likely to use CAM. This suggests that those who do not receive education from health professionals may be more likely to seek alternative treatments. This should be taken into consideration as it may cause patients to make the wrong choice.

## Suggestions

Complementary and alternative medicine involves applicable methods. However, healthcare professionals and the government should collaborate to integrate CAM therapies with modern treatment techniques. We should ensure that nurses train diabetic patients about their disease to help them make the right choices regarding CAM therapies.
